# 

**Published:** 1886-11-27

**Authors:** 


### CONTENTS.

**The Provision for Nursing Fevers in London** 139

**Words of Consolation.**—IX. Hope - - - - 140

**Hospital Support a Public Duty.**—By the Most Noble the Marquis of Salisbury, K.G. - - - - 141

**Hospital Co-operation and Mutual Help.**—By Sir Andrew Clark, Bart., M.D., F.R.S. - - - 142

**The Past, the Present, and the Future of Hospitals.**—By Sir Sydney Waterlow, Bart. 143

**Recent Novelties in Sick-room Appliances** - 144

**Flowers, Ferns, and Ward Decorations** - 145

**Notes and News.**—Miscellaneous.—Munificent Bequests.—"The Queen's Bed" at the North London Hospital.—The Children's Hospital at Paddington Green.—Lord Meath on the Maintenance of Hospitals.—The Netherfield Road Hospital.—Gifts to the Kilmarnock Infirmary.—Vacancies.—The St. John's Ambulance Association.—Increase of Pauper Lunatics.—The Stratford Dispensary.—The Great Northern Central Hospital.—Medical Classes at Aberdeen University.—Hospital Architects.—The Kyrle Society, etc. - - - - - 146

**Annotations.**—Books and Periodicals.—Who is Responsible?—Eight Years an Out-Patient.—Lunatics and Doctors.—Take Care of your False Teeth - - 149

**The Quilter Prizes** - - - - - - 150

**Willie:** A True Story - - - - - - 150

**Difficult Cases.**—A Case of Paralysis - - - 152

**Reviews and Notices.**—Ambulance Lectures - - 152

**Concerning Medical Students** - - - 153

**The Literary Prizes** - - - - - 153

**In- and Out-Patients' Hospital Diary** - - 154

**The Children's Corner.**—Puzzles, Answers, etc. - vi

**The Hospital Charity Box.**—Prizes and Results - vi

